# Insights into the Development of the Adult Leydig Cell Lineage from Stem Leydig Cells

**DOI:** 10.3389/fphys.2017.00430

**Published:** 2017-06-28

**Authors:** Leping Ye, Xiaoheng Li, Linxi Li, Haolin Chen, Ren-Shan Ge

**Affiliations:** The Second Affiliated Hospital and Yuying Children's Hospital of Wenzhou Medical UniversityWenzhou, China

**Keywords:** Leydig cells, testosterone, development, steroidogenic factor 1, Desert Hedgehog

## Abstract

Adult Leydig cells (ALCs) are the steroidogenic cells in the testes that produce testosterone. ALCs develop postnatally from a pool of stem cells, referred to as stem Leydig cells (SLCs). SLCs are spindle-shaped cells that lack steroidogenic cell markers, including luteinizing hormone (LH) receptor and 3β-hydroxysteroid dehydrogenase. The commitment of SLCs into the progenitor Leydig cells (PLCs), the first stage in the lineage, requires growth factors, including Dessert Hedgehog (DHH) and platelet-derived growth factor-AA. PLCs are still spindle-shaped, but become steroidogenic and produce mainly androsterone. The next transition in the lineage is from PLC to the immature Leydig cell (ILC). This transition requires LH, DHH, and androgen. ILCs are ovoid cells that are competent for producing a different form of androgen, androstanediol. The final stage in the developmental lineage is ALC. The transition to ALC involves the reduced expression of 5α-reductase 1, a step that is necessary to make the cells to produce testosterone as the final product. The transitions along the Leydig cell lineage are associated with the progressive down-regulation of the proliferative activity, and the up-regulation of steroidogenic capacity, with each step requiring unique regulatory signaling.

## Introduction

Adult Leydig cells (ALCs) are located in the interstitial compartment of the testis. The cells synthesize testosterone that is essential for the physiological functions of the male reproductive system. The pubertal development of ALCs is required for the initiation and maintenance of spermatogenesis as well as for the promotion of male secondary sexual characteristics. ALC originates from an undifferentiated stem cell, called stem Leydig cell (SLC). In rodents, two independent Leydig cell (LC) populations develop sequentially in the testis, one during the fetal period and the other during the puberty (Chen et al., 2017).

**Figure 1:**
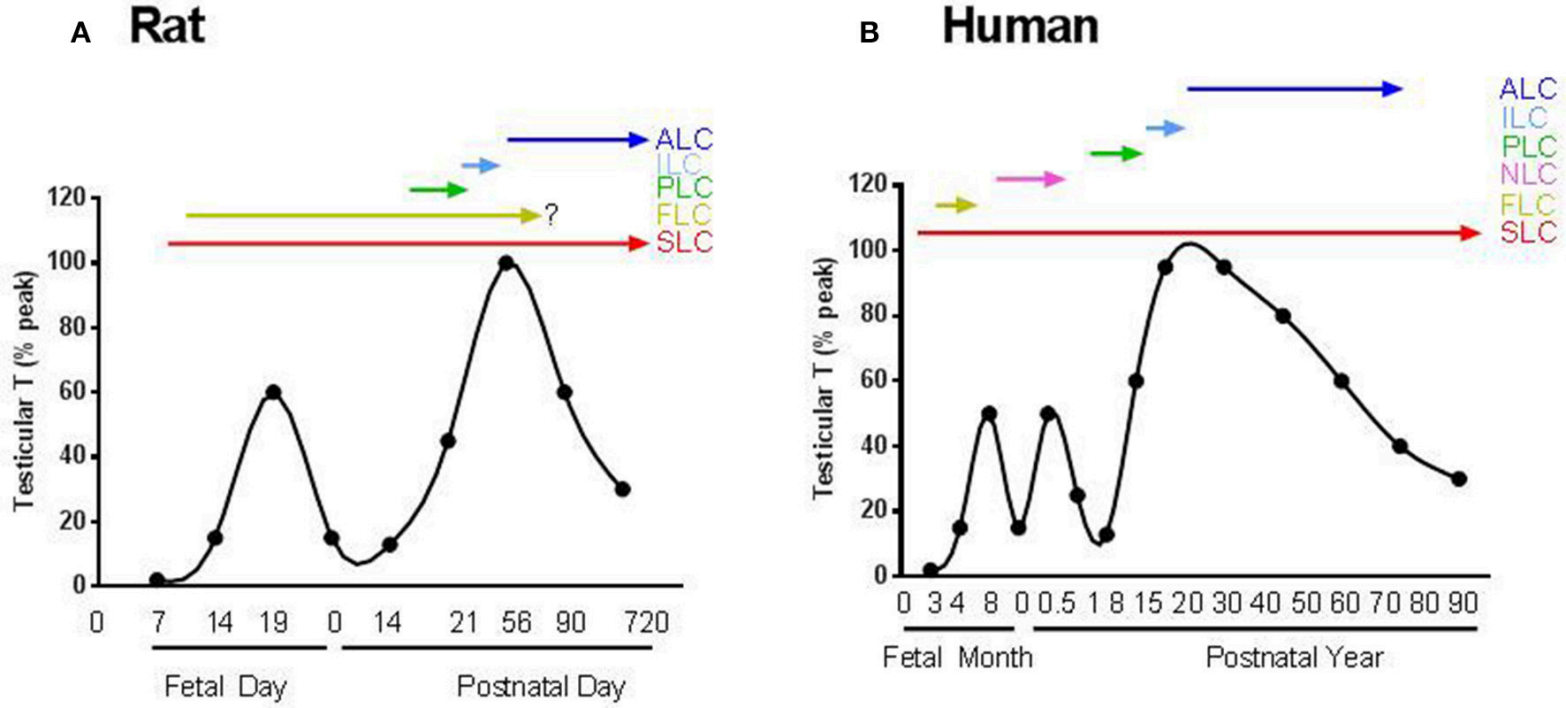
The scheme for the androgen production and possible involvement of Leydig cells in the life span. **(A)** Rat; **(B)** Human. SLC, FLC, PLC, ILC, ALC, and NLC represent stem, fetal, progenitor, immature, adult and neonatal Leydig cells, respectively. There are two androgen peaks for rats and three androgen peaks for human.

In mice and rats, the first generation, fetal Leydig cells (FLCs), develop between the testis cords on gestational days 11–12 (Figure 1A; Huhtaniemi and Pelliniemi, 1992; Yao et al., 2002). FLCs are differentiated from the WT1+ somatic progenitor pools in the gonadal primordium, which may also serve as SLCs for the adult Leydig cells (Liu et al., 2016). Once developed, FLCs become the terminally differentiated cells. They have the capacity to produce androstenedione and insulin-like factor 3 (INSL3) just prior to birth (Habert and Brignaschi, 1991; Zimmermann et al., 1999; Adham et al., 2000; O'Shaughnessy et al., 2000; Shima et al., 2013). Because the Sertoli cells contain 17β-hydroxysteroid dehydrogenase 3, the androstenedione produced by FLCs is further converted into testosterone (Shima et al., 2013). The androgen produced by FLCs is crucial for the development of male reproductive tract and the descent of the testis (Huhtaniemi and Pelliniemi, 1992). INSL3 is a critical factor for initiating the testis descent process (Adham et al., 2000).

Rodent FLCs develop independently from luteinizing hormone (LH) since the cells are well developed even in the absence of LH or their receptors (Lei et al., 2001; Zhang et al., 2001; Ma et al., 2004). Rodent FLCs remain in the testicular interstitium after birth but may involute gradually, with only a few persisting in the adult testis (Kerr and Knell, 1988). There is still controversy about whether FLCs ultimately die, dedifferentiate or survive in adult testis (Huhtaniemi and Pelliniemi, 1992; Wen et al., 2016). A recent study showed that FLCs indeed persisted in adult mouse testis (Shima et al., 2015). Even if they were survived through the adulthood, the contribution to androgen production by these FLC could be minimal because the relative number is low compared to adult population (Kerr and Knell, 1988).

In rodents, the first stage in ALC lineage that have committed from SLC is the progenitor Leydig cell (PLC) (Shan and Hardy, 1992). PLCs are first evident in rat testis on neonatal day 11 by their expressions of steroidogenic enzymes, including cytochrome P450 cholesterol side chain cleavage enzyme (CYP11A1), 17α-hydroxylase/20-lyase (CYP17A1), 3β-hydroxysteroid dehydrogenase (HSD3B) and LH receptor (LHCGR) (Figure 1A; Ariyaratne et al., 2000). By postnatal day 21, PLC reached the maximal level in the testis. By postnatal day 35, PLCs are completely converted into ILCs, and by day 56, most of ILCs become ALCs (Chen et al., 2009).

In the primate and human testes, there is another generation of LCs (Figure 1B), which develop and involute for a very short period of time during the neonatal stage (Nistal et al., 1986; Prince, 1990, 1992, 2001). The origin of this generation of neonatal LCs in human and other primates is still unclear. Unlike rodents, androgen production by FLCs in primates and human are highly relied on the placenta-secreted chorionic gonadotropin (Nistal et al., 1986). When chorionic gonadotropin level decreases in the second trimester during human pregnancy, FLC number reduces sharply (Codesal et al., 1990). In human, due to the transient activation of the hypothalamic-pituitary-testicular axis during the first 6 months after birth, the second generation of LCs develops which increases androgen production significantly and thereafter the cells either involute or dedifferentiate because the hypothalamic-pituitary-testicular axis becomes inactive again (Prince, 1990). This quiescence in LC activity and androgen production lasts for years until the beginning of the development of the third generation of LC, ALC. Although the exact physiological functions of this brief testosterone surge by neonatal LCs in humans are not clear, it is hypothesized that it might be necessary for imprinting various cell types in androgen dependent organs, including the brain, to insure their proper responses to androgen by the adult (Svechnikov et al., 2010). For example, it was found recently that in adult female, there is a central pattern generator (CPG) in brain that specifically controls parturition and the milk-ejection reflex. This CPG is active in both male and female neonates, but is inactivated in males after the first week of life by the neonatal testosterone pulse. This suggests that the neonatal LCs are important for gender-specific perinatal programming (Israel et al., 2014).

The fetal and adult populations of LCs in rodents have distinct morphological and functional differences: For example, FLCs have abundant lipid droplets and are encased by several layers of collagen-enriched membrane in clusters (Huhtaniemi and Pelliniemi, 1992) and they develop independent of LH (Zhang et al., 2001), while ALCs have few lipid droplets and do not form clusters and they becomes desensitized when they are exposed to high concentration of LH due to the presence of an inhibitory guanine nucleotide-binding protein, which does not exist in FLCs (Warren et al., 1984; Eskola et al., 1994). However, a recent lineage tracing study in mouse shows that both FLCs and ALCs develop from a common Wilms tumor protein 1 (Wt1) positive precursor cells (Liu et al., 2016). In this review, we will mainly focus on the development of ALC population.

## Adult leydig cell ontogeny

Our understanding of the development of ALC was mostly coming from studies of rodents (rat and mice). Therefore, in this review, we will focus on the results from these two species, with occasional mentions of other species if necessary. In rats and mice, the ALC lineage can be divided into four distinct stages: stem, progenitor, immature, and adult (Figure 1 and Table 1).

**Table 1:** The summary of cell properties in the adult Leydig cell lineage.

		**Leydig cell lineage**			
	**References**	**Stem**	**Progenitor**	**Immature**	**Adult**
**MORPHOLOGY**					
Shape	Shan et al., 1993	Spindle	Spindle	Ovoid	Round
Lipid droplets	Shan et al., 1993	None	Some	Many	Few
Endoplasmic reticulum	Shan et al., 1993	Few	Few	Advanced	Advanced
Mitochondria	Shan et al., 1993	Some	Some	Medium	Many
**PROLIFERATION**					
Self-renewal	Ge et al., 2006	Yes	None	None	None
Divisions	Ge and Hardy, 1997	Slowly	Highly	Once	None
Cyclin A2	Ge and Hardy, 1997	High	High	Medium	None
**BIOCHEMICAL PROPERTIES**					
Androgen	Ge and Hardy, 1998	None	Androsterone	Androstanediol	Testosterone
**BIOMARKERS**					
Nestin	Davidoff et al., 2004; Ge et al., 2006; Jiang et al., 2014	High	None	None	None
CD90	Li et al., 2016	High	None	None	None
CD51	Jiang et al., 2014; Zang et al., 2017	High	None	None	None
PDGFRA	Ge et al., 2006; Odeh et al., 2014	High	Medium	High	High
PDGFRB	Odeh et al., 2014	High	None	None	None
Notch2	Stanley et al., 2011	High	None	None	None
Frizzled1	Stanley et al., 2011	High	None	None	None
NR2F2	Kilcoyne et al., 2014	High	Medium	None	None
NR3C4	Kilcoyne et al., 2014	High	High	High	Low
CYP11A1	Ge and Hardy, 1998	None	Low	Medium	High
HSD3B1	Ge and Hardy, 1998	None	Low	High	High
CYP17A1	Ge and Hardy, 1998	None	Low	Medium	High
HSD17B3	Ge and Hardy, 1998	None	None	Medium	High
SRD5A1	Ge and Hardy, 1998	None	High	High	None
AKR1C9	Ge and Hardy, 1998	None	High	Medium	Low
HSD11B1	Phillips et al., 1989; Ge et al., 1997b	None	None	Medium	High
CYP2A1	Hu et al., 2010a	None	None	None	High

### Stem leydig cells (SLCs)

Until recently, a fundamental unanswered question about ALC development is whether these cells ultimately come from a single source of undifferentiated self-renewing SLCs. SLC should be defined to have the following three characteristics: (1) the capacity to self-renew for an extend period of time; (2) the potential to commit a lineage and expand the progenitor pool and differentiate to give rise the large number of progenies; and (3) the ability to move back to niche when transplanted back to the home organ (homing). SLCs are expected to be present in the testis in small numbers at adulthood and to be maintained by self-renewing. Furthermore, one progeny after an SLC division would be expected to undergo commitment into the LC lineage. The committed SLC, or PLC, amplifies its number through rapid cell divisions so as to create a pool of cells that are necessary to establish a right size of ALC population. It has been noted for some time that the postnatal rat testis contains spindle-shaped cells with few mitochondria or smooth endoplasmic reticulum that surround the seminiferous tubules and/or testicular blood vessels (Hardy et al., 1989). Cell dynamic analysis of the various populations in the interstitial compartment during the pubertal period suggested strongly that this spindle-shaped mesenchymal-like population may be the precursor of ALC (Hardy et al., 1989).

We isolated these cells by Percoll density-gradient centrifugation followed by immune-selections (Ge et al., 2006). Based on the 3 characteristics listed above, it was confirmed that these cells might be indeed the long-searched SLC. First, they express some stem cell markers, including Nestin and platelet-derived growth factor α receptor (PDGFRA) but do not express the LC lineage biomarkers, such as LHCGR, CYP11A1, HSD3B1 or CYP17A1 (Table 1; Ge et al., 2006). The cells can also proliferate, differentiate into the cells that can produce testosterone, and are capable of cloning (homing) in the testicular niche if they are transplanted back to the testis (Ge et al., 2006).

Various efforts were tried to find a specific protein marker to identify these cells from the rest of testicular cells (Chen et al., 2017). PDGFAA can specifically bind to PDGFRA to exert its signaling (Mariani et al., 2002). In the testis of mice that lack of PDGFAA no ALCs are developed, suggesting that PDGFRA signaling is critical for SLC function (Gnessi et al., 2000). The SLCs also express some levels of platelet-derived growth factor β receptor (PDGFRB), which is responsive to platelet-derived growth factor-BB, and the expression of this receptor disappears when SLCs enter the LC lineage (Stanley et al., 2012; Odeh et al., 2014).

In addition to PDGFRA, Nestin is another protein that may be served as the marker. nestin-positive SLCs contribute to the LC regeneration in the LC-depleted testis (Davidoff et al., 2004) that were induced by ethane dimethane sulfonate, a drug that specifically kills LCs (Teerds et al., 1988, 1989). In mouse testis, the nestin-positive SLCs can also proliferate, differentiate into the LC lineage *in vitro* and clone in the interstitial niche if they are transplanted back to the testis (Table 1; Jiang et al., 2014). Interestingly, nestin-positive SLCs also express CD51, a biomarker for the mesenchymal stem cells (Rux et al., 2016). Like nestin-positive cells, CD51-positive cells are also able to self-renew and differentiate into the multiple mesenchymal cell lineages and ALCs *in vitro*, as well as to clone back in the testicular niche (Table 1; Zang et al., 2017). This suggests that CD51-positive SLCs are multipotent. In addition to CD51, another biomarker for the mesenchymal stem cells (Paulini et al., 2016), CD90, may also be used to isolate the peritubular-associated SLC from the adult rat testis (Li et al., 2016). Interestingly, the CD90-positive cells can proliferate and differentiate into LC lineage *in vitro* in the absence of LH. The fact that these cells can be induced to differentiate into Leydig cells with Desert hedgehog (DHH), in the absence of other factors, including LH, suggests strongly that DHH may be the important SLC commitment factor that is necessary for the differentiation of SLC into Leydig lineage (Li et al., 2016).

Another biomarker of SLCs could be chicken ovalbumin upstream promoter transcription factor II (NR2F2 or COUP-TFII). Using lineage tracing analysis, it is found that NR2F2-positive cells can differentiate into ALCs (Table 1; Kilcoyne et al., 2014). Conditional knockout of NR2F2 during the pre-pubertal period prevented the formation of ALC population (Qin et al., 2008), suggesting that NR2F2-positive cells are critical seed cells for LC development. SLCs, judged by the expression of NR2F2, are present in the interstitium during the whole lifespan (Figure 1) and these cells are abundant during the neonatal and pre-pubertal periods (Kilcoyne et al., 2014).

### Progenitor leydig cells (PLCs)

In rat testis, PLC, the earliest identifiable cell stage in the differentiated LC lineage, first appears on postnatal day 11 (Ariyaratne et al., 2000). PLC is a small spindle-shaped cell that is morphologically similar to the undifferentiated SLC from which it is derived but contains LC markers, such as the steroidogenic enzymes CYP11A1, HSD3B1, and CYP17A1 (Shan et al., 1993). On postnatal day 12, PLCs also begin to express a truncated LHCGR (Figure 1A; Ge and Hardy, 2007).

PLCs may be called as amplifying cells because they have a high proliferative capacity and they express very higher levels of cyclin A2, a somatic cell cycle protein (Ge and Hardy, 1997). Additional cell cycle regulatory proteins, including cyclin-dependent kinase 2, cyclin-dependent kinase 25, cyclin B, cyclin C, cyclin D, and cyclin E are also abundant in PLCs (Ge et al., 2005; Stanley et al., 2011). PLCs retain the stem cell markers, PDGFRA, leukemia inhibitory factor receptor, and c-Kit (Ge et al., 2005; Stanley et al., 2011). Although CYP11A1, HSD3B, and CYP17A1 all appear in PLCs of wild-type mice, PLCs in the LHCGR knockout mouse is only positive for HSD3B but negative for both CYP11A1 and CYP17A1 (Zhang et al., 2004), suggesting that HSD3B may appear earlier than other steroidogenic proteins and therefore can be used as a better biomarker for the cells during the transition from SLCs into PLCs.

**Figure 2:**
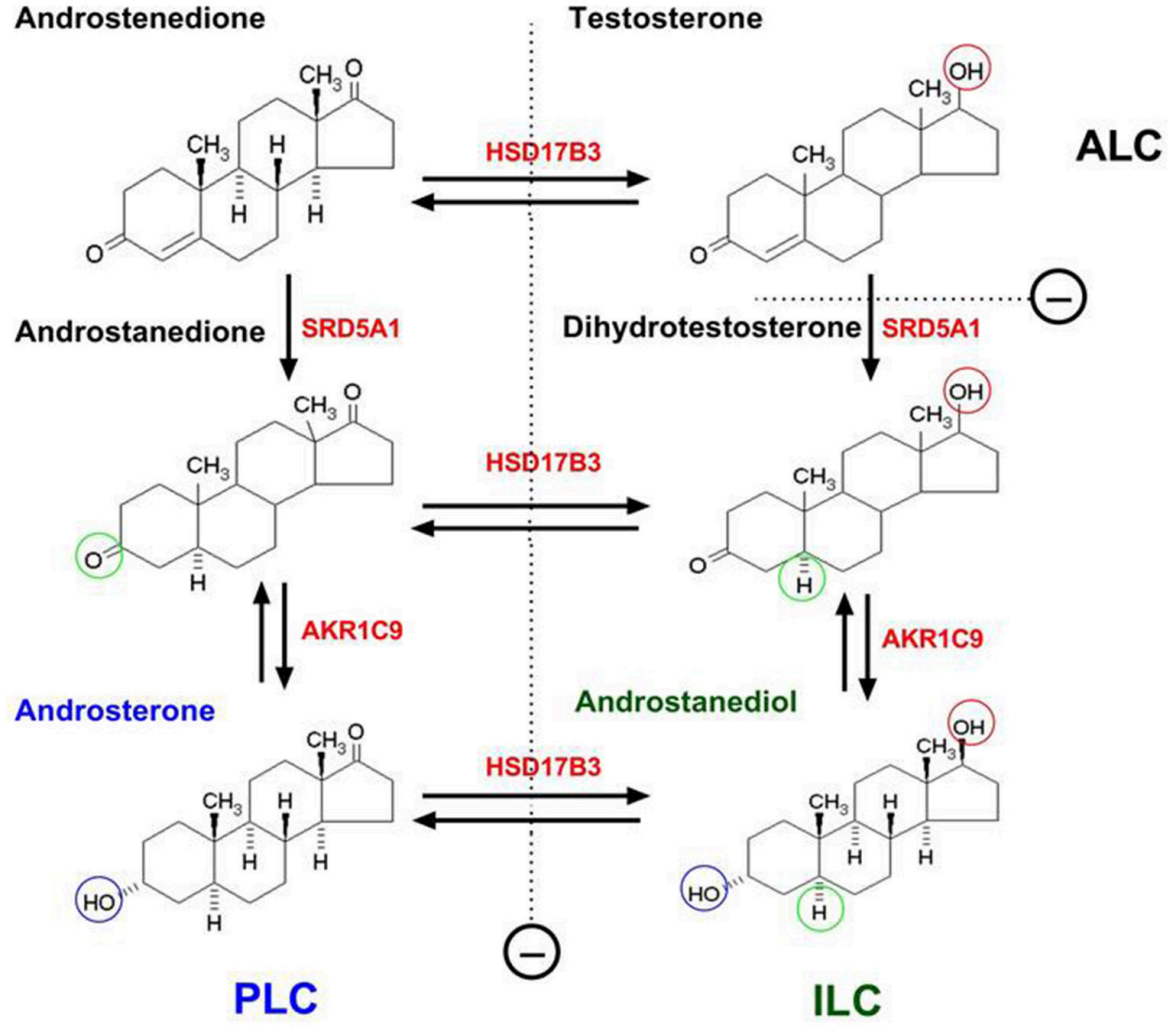
The difference of progenitor, immature and adult Leydig cells in the products of androgen in rats due to their differential expressions of steroidogenic enzymes. PLC, ILC, and ALC represent progenitor, immature, and adult Leydig cells, respectively. PLC lacks of 17β-hydroxysteroid dehydrogenase 3 (HSD17B3) but contains higher levels of 5α-reductase 1 (SRD5A1) and 3α-hydroxysteroid dehydrogenase (AKR1C9), thus producing primarily androsterone. ILC begins to express HSD17B3 and also contains SRD5A1 and AKR1C9, thus producing predominantly androstanediol. ALC secretes mainly testosterone due to the silence of SRD5A1. SRD5A1 is a unidirectional enzyme. Other steroidogenic enzymes are bidirectional.

PLCs do not express 17β-hydroxysteroid dehydrogenase 3 (HSD17B3), the critical enzyme to catalyze the formation of testosterone in the last step of steroidogenic pathway (Ge and Hardy, 1998). However, PLCs express high levels of androgen-metabolizing enzymes, 5α-reductase 1 (SRD5A1) and 3α-hydroxysteroid dehydrogenase (AKR1C9) (Ge and Hardy, 1998; Viger et al., 2005). Although PLCs have some potential to make androgens, they cannot make testosterone because of lacking HSD17B3 (Ge and Hardy, 1998). Thus, the androstenedione, formed after the sequential catalysis by three enzymes (CYP11A1, HSD3B, and CYP17A1) is metabolized into androstanedione by SRD5A1 and further into androsterone by AKR1C9, which is secreted as the end product of the cells (Figure 2; Ge and Hardy, 1998).

As they develop, PLCs enlarge the size and become ovoid-shaped (Benton et al., 1995). Their mitotic capacities are reduced when they acquire some of the differentiated functions of mature cells in the LC lineage (Ge and Hardy, 1997, 1998). Mouse PLCs are identified in the testis on postnatal day 10 (Baker et al., 1996; Hu et al., 2010b). Mouse PLCs also have high proliferative capacities to expand the cell pool in order to establish a right size of ALC eventually (Baker et al., 1996; Hu et al., 2010b).

### Immature leydig cells (ILCs)

The second transition in the lineage is from PLCs into ILCs. ILC is most commonly seen in rat testis from days 28 to 35 postpartum (Figure 1; Hardy et al., 1989). The cell is ovoid-shaped and appears to have more advanced smooth endoplasmic reticulum than PLC (Shan et al., 1993). In the rat, ILCs contain numerous lipid droplets (Shan and Hardy, 1992). The activities of CYP11A1, HSD3B1, and CYP17A1 increase in ILCs as they matures from PLCs (Ge and Hardy, 1998). ILCs begin to express HSD17B3 and therefore they can make testosterone from androstenedione (Ge and Hardy, 1998). However, ILCs still have higher levels of SRD5A1 (Viger and Robaire, 1995) and AKR1C9 (Shan et al., 1993), thus the formed testosterone by HSD17B3 is quickly metabolized into dihydrotestosterone by SRD5A1 and further into androstanediol by AKR1C9. The latter is secreted into the circulation as the final product of the cells (Figure 2; Ge and Hardy, 1998).

At this stage, a retinol dehydrogenase 2, which has 3α-oxidative activity that can convert androstanediol back to dihydrotestosterone (Hardy et al., 2000), also appears in ILCs. This enzyme is absent in PLCs (Ge et al., 1999). Interestingly, ILCs also begin to express 11β-hydroxysteroid dehydrogenase 1 (HSD11B1) on postnatal day 28 (Phillips et al., 1989). HSD11B1 is an oxidoreductase that controls local levels of glucocorticoid (Agarwal et al., 1989). ILCs in mouse testis seems also to express SRD5A1 and AKR1C9 and produces androstanediol during puberty (Wang and Hardy, 2004). Although it is clear that different stages of developing LCs produce different forms of androgens, it is still unclear whether there is any physiological significance behind it.

Around postnatal day 35, the number of ILCs is about 50% of ALCs in the adult testis (Hardy et al., 1989). This suggests that ILCs, on average, divide at least once in order to establish a right size of ALC population in adulthood. Indeed, even with reduced levels of cell cycle regulating proteins, including cyclin A2, cyclin B, cyclin C, and cyclin E (Ge et al., 2005; Stanley et al., 2011), ILCs still have maintained some proliferative capacity (Ge and Hardy, 1997).

### Adult leydig cells (ALCs)

ALCS in rat testis is formed on postnatal days 49–56 (Figure 1; Hardy et al., 1989). ALCs are irregularly round and are larger than ILCs (Shan et al., 1993). ALCs have well developed smooth endoplasmic reticulum and more mitochondria than ILCs and have increased expressions of CYP11A1, HSD3B, CYP17A1, and HSD17B3. At this stage, the SRD5A1 expression is silenced (Viger and Robaire, 1995), which leads to testosterone as the major product of the cells (Figure 2; Ge and Hardy, 1998). Testosterone is synthesized from cholesterol in ALCs in a multistep process catalyzed by four enzymes sequentially: CYP11A1, HSD3B, CYP17A1, and HSD17B3 (Payne and O'Shaughnessy, 1996). The transportation of cholesterol from the cytosol into the inner membrane needs steroidogenic acute regulatory protein (StAR) (Clark et al., 1995). The expression of this protein is activated by LH via cAMP signaling pathway (Stocco, 2001). In addition to steroidogenic enzymes that are directly responsible for testosterone production, HSD11B1 is also highly expressed by ALC (Ge et al., 1997b). This enzyme may behave primarily as a protecting protein to prevent the cells from the negative effects of excessive glucocorticoid exposure. HSD11B1 is a bi-directional enzyme that can convert glucocorticoid between the active/inactive states. The directions are regulated by another enzyme, hexose-6-phosphate dehydrogenase. Since this regulatory enzyme is very low in ALCs (Ge et al., 1997a; Li et al., 2015) HSD11B1 mainly functions as an oxidase to metabolize glucocorticoid into an inactive form. Also, in rat ALCs, a CYP2A1 begins to express and it can catalyze testosterone into 7α-hydroxytestosterone, which also involves in the regulation of HSD11B1 activities (Hu et al., 2010a). In the rat testis, ALCs rarely, if ever, divide; the ALC number is maintained relative stable at about 25 million cells per testis (Hardy et al., 1989).

## Regulation of leydig cell development

**Figure 3:**
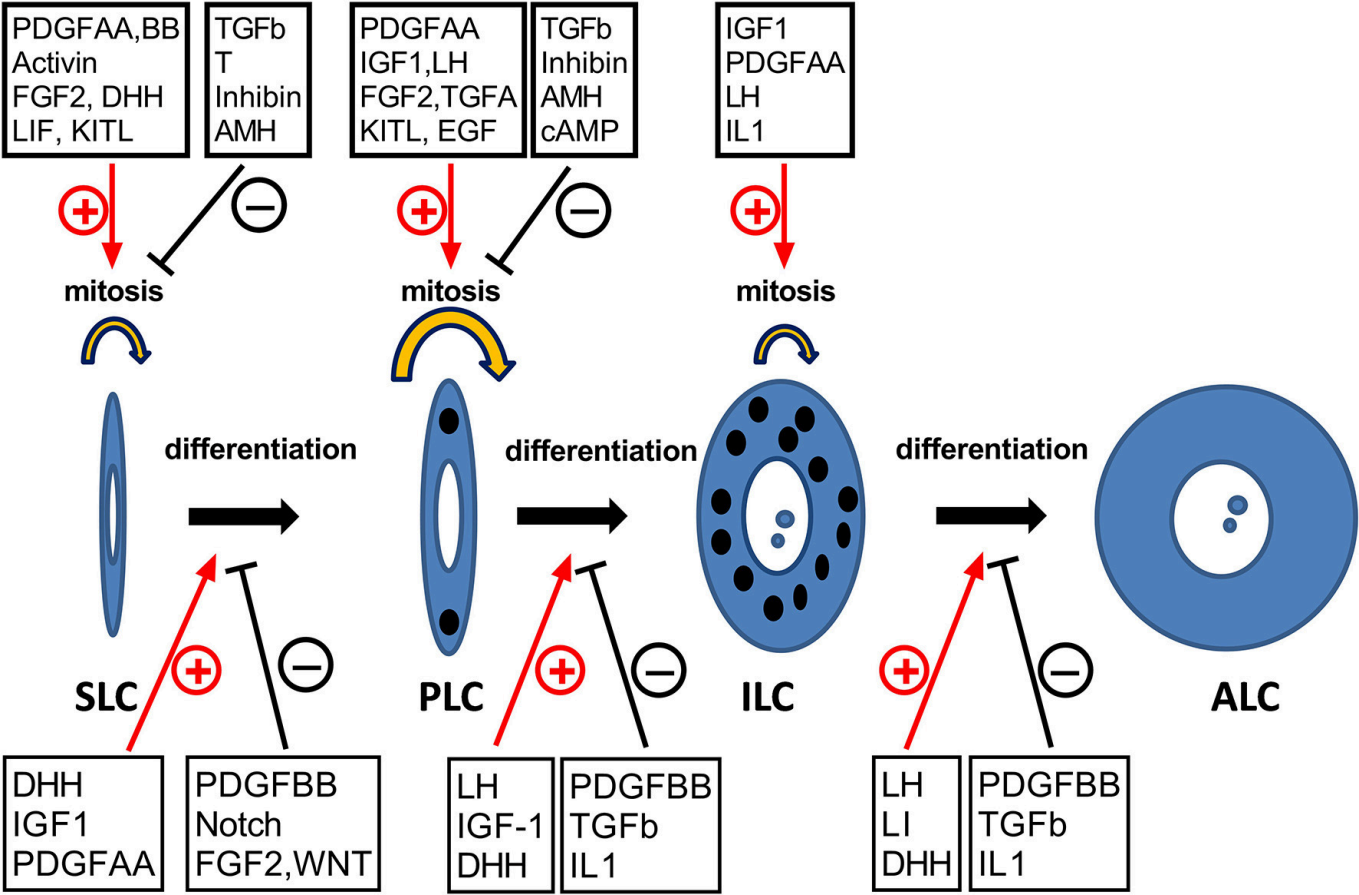
The hormones and growth factors that may potentially regulate the development of Leydig cells. SLC, PLC, ILC, and ALC represent stem, progenitor, immature, and adult Leydig cells, respectively. ⊕, stimulation; θ, inhibition.

The proliferation and differentiation of cells in the LC lineage are regulated by multiple growth factors and hormones (Figure 3 and Table 2). These factors act via various signaling pathway, positively or negatively controlling the progression of each of developmental LC stages.

**Table 2:** Regulatory factors and hormones for Leydig cell development.

		**Leydig cell lineage**			
	**References**	**Stem (SLC)**	**Progenitor (PLC)**	**Immature (ILC)**	**Adult (ALC)**
**GROWTH FACTORS**					
Leukemia inhibitory factor	Ge et al., 2006; Wang et al., 2016	SLC mitosis↑ SLC → PLC(↔)	ND	ILC → ALC↑	Steroidogenesis(↔)
Desert Hedgehog	Clark et al., 2000; Park et al., 2007; Li et al., 2016	SLC mitosis↑ SLC → PLC↑	PLC mitosis↑ PLC → ILC↑	ILC mitosis↑ ILC → ALC↑	Steroidogenesis↑
Platelet-derived growth factor-AA	Schmahl et al., 2008; Odeh et al., 2014; Li et al., 2016	SLC mitosis↑ SLC → PLC↑	PLC mitosis↑ PLC → ILC↑	ILC mitosis↑ ILC → ALC↑	Steroidogenesis↑
Platelet-derived growth factor-BB	Odeh et al., 2014; Li et al., 2016	SLC mitosis↑ SLC → PLC↓	PLC mitosis↑ PLC → ILC↓	ILC → ALC↓	Steroidogenesis↓
Kit ligand	Rothschild et al., 2003; Liu et al., 2017	SLC mitosis↑ SLC → PLC↑	PLC mitosis↑ PLC → ILC↑	ILC mitosis↑ ILC → ALC↑	Steroidogenesis↑
Insulin-like growth factor 1	Baker et al., 1996; Hu et al., 2010b	SLC mitosis (↔) SLC → PLC↑	PLC mitosis↑ PLC → ILC↑	ILC mitosis↑ ILC → ALC↑	Steroidogenesis↑
Transforming growth factor ↑	Lin et al., 1987; Park et al., 2014; Li et al., 2016	SLC mitosis↓ SLC → PLC↓	PLC mitosis↓ PLC → ILC↓	ILC mitosis↓ ILC → ALC↓	Steroidogenesis↓
Anti-Müllerian hormone	Lyet et al., 1995; Sriraman et al., 2001; Salva et al., 2004	SLC mitosis↓ SLC → PLC↓	PLC mitosis↓	ILC mitosis↓	Steroidogenesis↓
Activin A	Lin et al., 1989; Li et al., 2017	SLC mitosis↑ SLC → PLC↓	PLC mitosis↑ PLC → ILC↓	ILC mitosis↑ ILC → ALC↓	Steroidogenesis↓
Fibroblast growth factor 2	Lin et al., 1989; Li et al., 2017	SLC mitosis (↔) SLC → PLC↑	PLC mitosis↑ PLC → ILC↑	ILC mitosis↑ ILC → ALC↑	Steroidogenesis↑
LH	Lin et al., 1989; Lei et al., 2001; Zhang et al., 2001; Li et al., 2017	SLC mitosis (↔) SLC → PLC(↔)	PLC mitosis↑ PLC → ILC↑	ILC mitosis↑ ILC → ALC↑	Steroidogenesis↑
Androgen	Lin et al., 1989; Kilcoyne et al., 2014; Li et al., 2017	SLC mitosis↑ SLC → PLC↓	PLC → ILC↑	ILC mitosis↑ ILC → ALC↓	Steroidogenesis↓
Notch ligand	Tang et al., 2008; Defalco et al., 2013; Li et al., 2016	SLC mitosis ↑ SLC → PLC↓	PLC mitosis↑ PLC → ILC↓	ILC mitosis↑ ILC → ALC(↔)	Steroidogenesis↓

*↓, inhibition; ↑, stimulation; ↔, No effect*.

### Transcription factors

Several transcription factors are critical for LC development. One of these factors is steroidogenic factor 1 (SF1, also known as NR5A1 or AD4BP). NR5A1 is a common transcription factor for the somatic stem cells in the gonads (Sadovsky et al., 1995), the adrenals (Gut et al., 2005), and the pituitary (Zhao et al., 2001). Inactivation of NR5A1 causes gonadal agenesis and loss of all somatic cell lineages in the gonad, including LCs (Sadovsky et al., 1995; Zhao et al., 2001). The importance of NR5A1 in LC development is also evident by the observations that forced expression of this gene, by itself, can convert stem cells of other organs and fibroblasts into LC lineage by promoting the expression of LHCGR and other steroidogenic enzymes (Cyp11a1, Hsd3b, Cyp17a1, and Hsd17b3; Yang et al., 2017).

GATA4 is a transcription factor with a double zinc finger binding domain that recognizes the GATA motif in the promoters of the target genes (Tevosian, 2014). GATA4 is expressed in SLCs and is a key positive transcriptional regulator of genes involved in steroid biosynthesis, including transcriptional factor NR5A1 (Hu et al., 2013). GATA4 is required for the formation of genital ridge in mice (Hu et al., 2013). One of the functions of GATA4 could be to induce NR5A1 expression.

Different from NR5A1 and GATA4, NR2F2 seems to play a very narrow and specific role in LC development. Without it, the maturation of PLC to ALC is inhibited. Knockout of NR2F2 on postnatal day 14 does not influence the formation of PLCs but significantly blocks the transition of PLCs into ILCs as judged by failed changes in cell morphology and by remarkable reductions in the expressions of steroidogenic enzymes such as *Cyp11a1, Hsd3b*, and *Cyp17a1* (Qin et al., 2008).

NR4A1 (also known as Nur77, NGFI-B, or TR3) is another major nuclear receptor transcription factors involved in the regulation of LC development and steroidogenesis (Zhang and Mellon, 1997). NR4A1-binding regions are identified within the promoters of several LC steroidogenic-related genes, including rat *Cyp17a1* (Zhang and Mellon, 1997), mouse *Star* (Martin et al., 2008), and human *HSD3B* (Martin and Tremblay, 2005). Interestingly, NR4A1 is regulated by LH, which may mediate the roles of LH in the development of LCs (Song et al., 2001).

### Leukemia inhibitor factor (LIF)

In addition to transcriptional factors, multiple external factors are identified that may regulate SLC commitment and progression. One such factor is LIF, a member of the interleukin-6 cytokine family (You et al., 2017), which includes interleukin 11, interleukin 27, and ciliary neurotrophic factor. These factors bind to homo- or heteromeric receptors, of which they share a common gp130 subunit (Cheng et al., 1994; Moreno Frias et al., 2004). LIF receptor contains two subunits, gp130 and gp190 (Cheng et al., 1994; Moreno Frias et al., 2004). When LIF binds to gp190, the bound receptor causes the heterodimerization of gp130 and gp190 subunits, which subsequently induces a rapid activation of janus kinases, resulting in the phosphorylation of tyrosine residues of gp190 and gp130 (Golshan et al., 2014). These phosphorylated tyrosine residues activate signal transducer and activator of their target transcription families (Heinrich et al., 1998; Moser et al., 2001; Xi et al., 2012).

The expression level of LIF in rat fetal testis is low which is significantly increased during puberty and reaches the highest level around postnatal day 45 (Piquet-Pellorce et al., 2000). LIF is required for the self-renewal of embryonic stem cells (Wang et al., 2017), indicating that it may also be critical for the self-renewal of SLCs. In rat testis, LIF is first detected during gestation period. It is expressed predominantly by the peritubular cells surrounding the seminiferous tubules (Piquet-Pellorce et al., 2000), indicating that these LIF expressing cells may form a niche for SLCs since SLCs were also reported to reside on the surface of seminiferous tubules (Zhang et al., 2013; Odeh et al., 2014; Li et al., 2016). Indeed, LIF is capable of stimulating the proliferation of SLCs *in vitro* (Ge et al., 2006).

### Desert hedgehog (DHH)

The Hedgehog signaling pathway is involved in a number of developmental processes during embryogenesis (see review, Varjosalo and Taipale, 2008). Hedgehog factors have three members: DHH, Indian Hedgehog, and Sonic Hedgehog. These Hedgehogs induce a common signal transduction pathway via binding to a receptor complex consisting of patched receptors (Ptch1 and Ptch2) and Smoothened on the cell membrane (Yao et al., 2002; Park et al., 2007). This signaling pathway involves three transcription factors Gli1, Gli2, and Gli3 (Ingham and Mcmahon, 2001). Gli1 acts as a transcriptional activator while Gli2 and Gli3 are needed to be processed by proteasomes, thus repressing target gene transcription (Varjosalo and Taipale, 2008). Patched inhibits Smoothened activity by preventing its accumulation within cilia (Rohatgi et al., 2007). Binding of Hedgehog to Patch releases its inhibitory effect on Smoothened, allowing Smoothened to induce the downstream signaling related with Gli proteins (see review Varjosalo and Taipale, 2008).

In the testis, DHH is secreted by Sertoli cells after the activation of Sex-determining Region of Y-chromosome (SRY) and the expression persists into the adult (Bitgood et al., 1996). Ptch1 is present in the LC lineage (Clark et al., 2000). DHH-Ptch1 signaling seems to be critical for LC development. Rat testis with a null mutation of DHH gene (*Dhh*) lacks ALCs (Kawai et al., 2010). DHH may have similar functions for human SLC development because a patient with a homozygous mutation in *DHH* gene shows partial gonadal dysgenesis with the loss of ALCs (Werner et al., 2015). The development of ALCs is also defective in *Dhh* knockout male mice, and the mice are sterile (Clark et al., 2000). Loss of *Dhh* leads to a reduced number of FLCs, indicating that both FLCs and ALCs require DHH regulation (Yao et al., 2002). In *Dhh* knockout mice, the migration of fetal LC precursors from the mesonephros and their proliferation and survival are not affected, suggesting that DHH mainly affects the differentiation of fetal LC precursors to LC lineage (Yao et al., 2002).

Using rat seminiferous tubule culture system, we demonstrate that DHH is required for both proliferation and differentiation of SLCs into ALC lineage (Li et al., 2016). DHH alone is sufficient to induce the differentiation of CD90-positive SLCs isolated from the seminiferous tubules into LC lineage (Li et al., 2016). Although the mechanism by which DHH induces LC differentiation is still unknown, NR5A1 seems to be involved since DHH is able to stimulate the expressions of this transcriptional factor in Leydig cells (Yao et al., 2002; Barsoum et al., 2013). Ectopic activation of the Hedgehog signaling in the ovary also stimulated NR5A1 expression (Barsoum et al., 2013), which induced the formation of LC-like cells in the ovary, where LCs normally are absent. It is possible that DHH not only affects Leydig cell development, but also may play a role in the maintenance of steroidogenic function of ALC. Conditional knockout of *Wt1* gene in Sertoli cells in 2 month-old mice resulted in loss of ALC functions, as shown by a severe reduction in testosterone production and significant downregulations of steroidogenic enzymes and LHCGR, all possibly because of loss of *Dhh* expression, since the defects in steroidogenesis can be partially rescued by a hedgehog pathway agonist (Chen et al., 2014).

### Platelet-derived growth factors

Platelet-derived growth factors (PDGF) are a family of proteins that have been shown to exert mitogenic effects on undifferentiated mesenchyme and progenitor cell populations (Brennan et al., 2003). PDGF consist of several subunits as the homo- or heteromeric complex. For example, PDGF-A and PDGF-B subunits can form AA, AB, and BB dimers, which can bind to their respective receptors.

PDGF receptor α (PDGFRA) is expressed in the LC lineage cells, including the peritubularly located SLCs (Gnessi et al., 1995). The major ligand of PDGFRA is PDGF-AA (Mariani et al., 2002). It has been shown that ALCs do not differentiate in the *Pdgfa* knockout mice, suggesting that PDGF-AA signaling is critical for the SLC function (Gnessi et al., 2000). Interestingly, SLCs also express some levels of platelet-derived growth factor receptor β (PDGFRB) (Gnessi et al., 1995), whose nature ligand is PDGF-BB (Stanley et al., 2012). The expression of PDGFRB is silenced when SLCs enter the LC lineage in rats (Stanley et al., 2011; Odeh et al., 2014).

Two PDGF receptors-mediated signaling has similar and dissimilar actions in SLCs (Odeh et al., 2014; Li et al., 2016). Both significantly stimulate the proliferation of SLCs when they bind to their respective receptors (Li et al., 2016). PDGFRA signaling stimulates the differentiation of SLCs into the LC lineage when it binds to PDGF-AA (Odeh et al., 2014), while PDGFRB signaling inhibits the differentiation of SLCs into the LC lineage when it binds to PDGF-BB (Odeh et al., 2014). After the treatment of PDGF-BB, peritubular myoid cells are differentiated, indicating that SLCs are multipotent, being capable of committing into either the LC lineage or the myoid cell lineage, depending on the stimulating PDGF ligands (Odeh et al., 2014; unpublished observation).

The expression of PDGFRA is regulated by the transcription factors SP1 and SP3 (Bergeron et al., 2011). The downstream signaling of PDGFRA in regulating the commitment of SLCs into the LC lineage is still unclear. Two PDGF target genes (*Sgpl1* and *Plekha1*) have a significant role in the development of ALCs, since knockouts of either *Sgpl1* or *Plekha1* genes caused a phenotype similar to that of *Pdgfa* mutation, with a dramatic loss of ALC population (Schmahl et al., 2008). Sphingosine-1-phosphate lyase, encoded by *Sgpl1*, is an enzyme that irreversibly cleaves sphingosine-1-phosphate (Kihara et al., 2003), suggesting that sphingosine-1-phosphate may mediate the downstream signaling of PDGFRA in the LC development. *Plekha1* encodes a pleckstrin homology domain-containing adapter protein, which is localized in the plasma membrane, where it specifically binds to phosphatidylinositol 3,4-bisphosphate and thus possibly involved in the formation of signaling complexes in the plasma membrane (Dixon et al., 2011). PDGFRA is also important for FLC development because Pdgfrα^−/−^ male mice have reduced FLC numbers (Brennan et al., 2003).

### Kit ligand (KITL)

KIT ligand (also called stem cell factor) is a growth factor that can bind its receptor, KIT (c-kit) to activate its tyrosine kinase activity. Autophosphorylation of the receptor initiates signaling transduction cascades, including activation of PI3-kinase (PI3K) signaling by binding to a p85 subunit of PI3-kinase. In the testis, the KIT ligand is secreted by Sertoli cells. There are two forms of the ligand, KITL1 and KITL2, which are produced via alternatively spliced *Kitl* mRNAs. KITL1 is processed to produce soluble KIT ligand, and KITL2 is a membrane-bound factor (Minegishi et al., 2003). KIT is expressed by germ cells and SLCs (Rothschild et al., 2003). KIT ligand plays a critical role in the development of primordial germ cells. In mice with null mutation of either KIT or KIT ligand, primordial germ cells cannot proliferate and migrate, which leads to only a few primordial germ cells in the gonad (Augustowska et al., 2003a; Min et al., 2004). In mice with reduced KIT and KIT ligand, the postnatal spermatogenesis is blocked (Augustowska et al., 2003b). A mutation in KIT that lost its binding ability to PI3-kinase due to the substitution of tyrosine by phenylalanine, also caused severe deficiencies in spermatogenesis (Rothschild et al., 2003). These results indicate that KIT system is important to spermatogenesis.

In the newborn rat and mice testis, KIT is also expressed in the LC lineage, includingSLCs by postnatal day 5 (Manova et al., 1990; Sandlow et al., 1996). The role of KIT ligand in LC development is supported by both *in vivo* and *in vitro* studies. In KIT mutant mouse, LC has a reduced capacity of steroidogenesis (Rothschild et al., 2003). Using an *in vitro* culture system of seminiferous tubules from LC-depleted testis, it is found that KIT ligand can affect both proliferation and differentiation of the peritubularly located SLC. Interestingly, the two effects happened at different concentrations, with proliferation taking place at high concentrations (10 and 100 ng/ml) while differentiation taking place at low concentration (1 ng/ml) (Liu et al., 2017).

### Insulin-like growth factor 1 (IGF1)

Insulin-like growth factor 1 (IGF1), a factor with a similar structure to insulin, plays a very diversified effects cross our body. IGF1 is primarily secreted by the liver and is also produced locally by the target tissues. In the testis, IGF1 is secreted by peritubular cells, spermatocytes, and LCs (Handelsman et al., 1985; Lin et al., 1986a,b; Dombrowicz et al., 1992; Ha et al., 2016). The secretion of IGF1 in liver is regulated by growth factor. IGF1 initiates the intracellular signaling by binding its specific receptor and activating its tyrosine kinase activity (Yakar et al., 2002; Ha et al., 2016). IGF1 receptor is present in the LC lineage (Vannelli et al., 1988).

FLC seems not requiring IGF1 for its development since *Igf1* gene knockout does not affect FLC population in mice (Baker et al., 1996). However, the deletion of *Igf1* gene results in a significant reduction in testosterone production of the adult testis due to a reduced LC numbers (Baker et al., 1996). IGF1 is capable of stimulating the proliferation of PLCs and ILCs, and knockout *Igf1* completely abolishes their proliferations *in vivo* (Hu et al., 2010b). The replacement of IGF-1 to these null mice restored the LC population by increasing the proliferations of PLCs and ILCs (Hu et al., 2010b). Interestingly, IGF1 may not affect the proliferation of SLCs (Hu et al., 2010b).

In addition to cell proliferation, IGF1 also stimulates LC differentiation. Deletion of *Igf1* significantly delayed the maturation of ILCs to ALCs since the cells in the mutant adult testis still had characteristics of ILCs, with significantly decreased expression of CYP11A1, HSD3B, CYP17A1, and HSD17B3 and increased expression of SRD5A1 (Wang et al., 2003). LH can stimulate IGF1 secretion and upregulate IGF1 receptor expression in LCs (Lin et al., 1988; Cailleau et al., 1990; Nagpal et al., 1991) so it is possible that IGF1 may partially mediate the effect of LH in Leydig cell development (Khan et al., 1992b; Ge and Hardy, 1997). Replacement of IGF1 can also increase testosterone production in growth hormone deficient Snell dwarf mice, which have low circulating IGF1 levels (Dombrowicz et al., 1992).

### Transforming growth factor β1 (TGFb)

Transforming growth factor β1 (TGFb1) is a member of the TGFb superfamily. It exerts its signaling via binding to TGFb type II receptor and TGFb type I receptor (also termed activin receptor-like kinase-5, ALK5), both of which are serine/threonine kinase receptors (Goumans et al., 2002). Binding of TGFb to type II receptor induces the formation of heteromeric complexes with ALK5, within which type II receptor phosphorylates ALK5, turning on receptor kinase activity. Activation of ALK5 induces Smad2 and/or Smad3 phosphorylation at C-terminal serines, and the activated Smad2 and/or Smad3 forms a heterotrimeric complex with Smad4, which translocates to the nucleus for transcription regulation (Goumans et al., 2002).

Among the superfamily, TGFb is the most prominent type in the testis (Skinner and Moses, 1989). On postnatal day 21, about 50% of the PLCs express TGFb while ILCs from postnatal day 35 have undetectable level of expression (Teerds and Dorrington, 1993). TGFb inhibits both the proliferation and differentiation of rat SLCs on the surface of seminiferous tubules (Li et al., 2016). However, its effects on PLC proliferation seems to depend on the presence of other factors. For example, TGFb causes a small but significant increase in PLC proliferation when used by itself but inhibits the proliferations induced by transforming growth factor α or IGF1 (Khan et al., 1992b) when used with these factors together. TGFb also potently inhibits the steroidogenesis in PLCs (Khan et al., 1999). It represses LH-stimulated testosterone production in LCs through decreasing LHCGR and steroidogenesis-related genes such as *Star* and *Cyp17a1* (Le Roy et al., 1999). One of the possible mechanisms by which TGFb affect Leydig cells is through modifying nuclear factor NR4A1 activity. It was found that TGFb/ALK5/SMAD3 signaling can repressed the ability of NR4A1 transcription factor to bind the promoters of the target genes (Park et al., 2014).

### Anti-Müllerian hormone (AMH)

Anti-Müllerian hormone (AMH, also called Müllerian inhibiting substance) is another member of the TGFb superfamily. AMH is a hormone that inhibits the development of the Müllerian ducts in the male embryo to insure the development of the male reproductive system. Its productions are high in the fetal and neonatal testes (mainly by immature Sertoli cells) and the production declines by the puberty (Vigier et al., 1989; Racine et al., 1998). The AMH receptor type II (AMHR2) is a serine/threonine kinase receptor that is also expressed in the LC lineage (Racine et al., 1998). Overexpression of human AMH in mice reduced LC numbers in the testis (Lyet et al., 1995). In contrast, AMH and AMHR2 deficiency in mice increased LC numbers (Lyet et al., 1995; Racine et al., 1998) and AMH/AMHR2 double knockout in mice have caused LC hyperplasia (Mishina et al., 1996). Treatments of AMH *in vitro* and *in vivo* significantly inhibited LC steroidogenesis by downregulating Lhcgr and *Cyp17a1* expressions (Sriraman et al., 2001). AMH also inhibits the regeneration of LCs in ethane dimethane sulfonate-treated rats by inhibiting PLC proliferation and promoting LC apoptosis (Salva et al., 2004). Based on the studies of a transgenic mice model, it was found that AMH inhibited PLC proliferation by activating ALK3 (Wu et al., 2012).

### Activin and inhibin

Activin is also a member of the TGFb superfamily (Sharpe, 2006). Activin is a dimeric ligand, containing either homodimer or heterodimer of the 3 inhibin b subunits βA (encoded by *Inhba*), βB (encoded by *Inhbb*), or βC (encoded by *Inhbc*). Activin A (two βA subunits) acts via a heteromeric complex of receptor serine kinases, which include at least two type I (I and IB) and two type II (II and IIB) receptors (Sharpe, 2006). These receptors are transmembrane proteins, which contain an extracellular domain with the cysteine-rich region for the ligand-binding, a transmembrane domain, and a cytoplasmic domain with serine/threonine specificity for signaling. Type I receptors, including activin A receptor type I, are essential for signaling. Type II receptors are required for binding to activin A and for the expression of type I receptors. Type I and II receptors form a stable complex upon activin A binding, leading to phosphorylation of type I receptors by type II receptors (Sharpe, 2006). Activin A has been shown to be an important growth factor in the testis to regulate its functions (Hutson and Hasthorpe, 2005; Paul et al., 2005; Rey et al., 2005; Mendis-Handagama et al., 2006). As the product of FLCs, activin A regulates Sertoli cell mitosis and the testis cord expansion (Paul et al., 2005). Rat neonatal testis contains high number of SLCs (Chen et al., 2009) and a higher level of activin A is observed during this period (Kai et al., 2005). With the ALC development, activin A protein levels decrease while two activin signaling inhibitors, follistatin and BMP, and activin membrane-bound inhibitor increases (Borch et al., 2005). Follistatin increases in the mouse testis after postnatal day 4 (Trbovich et al., 2004). Activin A increases rat SLC and PLC proliferations. However, activin A may inhibit SLC differentiation to maintain their stemness (Li et al., 2017). Activin also inhibits ALC steroidogenesis (Lin et al., 1989).

Inhibin is another member of TGFb superfamily. The first component of the dimer ligand is a β subunit identical to the β subunit in activin while the second component is a more distantly-related α subunit (Burger and Igarashi, 1988). Inhibin is secreted by the Sertoli cells (Skinner et al., 1989). Interestingly, androgens can stimulate inhibin productions, which implies thatLCs can affect the development of themselves via inhibin (Meachem et al., 2001) since it was showed that inhibin can inhibit SLC proliferation (Li et al., 2017). Much less is known about the mechanism by which inhibin works. It may act via competing with activin for binding to activin receptors and/or binding to inhibin-specific receptors (Robertson et al., 1992).

### Fibroblast growth factor 2

Fibroblast growth factor 2 (FGF2) belongs to a heparin-binding growth factor family. It can affect multiple cell functions, including proliferation, migration, survival, and differentiation. The factor can affect diverse cell types and tissues (Eswarakumar et al., 2005). FGF2 is also identified in testis (Ueno et al., 1987). It is expressed in the LC lineage, and its level decreases with the progression of PLCs into ALCs (Ge et al., 2005). The involvement of FGF2 in the development and function of LCs could be anticipated, because of the mesenchymal origin of this lineage. FGF2 dramatically stimulates SLC proliferation while it inhibits its differentiation (Li et al., 2016). FGF2 stimulates the proliferation of SLCs in ethane dimethane sulfonate-treated LC regeneration model (Liu et al., 2014). FGF2 can downregulate NR5A1 expression which could be one of the machanisms that FGF2 inhibits the PLC differentiation. Although FGF2 can stimulate ILC steroidogenesis in the absence of LH, FGF2 has a biphasic effect on LH binding to its receptors (LHCGR) in ILCs, with low concentrations (0.1–1.0 ng/ml) inhibitory and high concentrations (10–100 ng/ml) stimulatory (Murono et al., 1993). The late may be mediated by FGF2 binding to a different receptor, heparan sulfate proteoglycans (Murono et al., 1993).

### Luteinizing hormone (LH)

Cells in Leydig cell lineage are the only cells that respond to LH in the testis. LH binds to the LHCGR in LCs, resulting in both acute- and trophic-effects. Acute-effects involve the mobilization and delivery of cholesterol to mitochondran to start the steroidogenesis. The trophic effects involve, increases in gene transcriptions and steroidogenic enzyme activities. Both effects are required for the maintenance of an optimal steroidogenesis in the cells (Dombrowicz et al., 1996). Without LH stimulation, LC steroidogenic enzyme activities are reduced. Although LH stimulation is required for LC development, it seems unlikely that the horomone is required for the commitment of SLC into the LC lineage because PLCs can be formed in Lhcgr knockout mouse testis (Zhang et al., 2004). LH seems to not affect FLC differentiation because FLCs are well developed in the Lhcgr- and Lhb- knockout mice (Ma et al., 2004; Zhang et al., 2004). However, studies of LHb and GnRH knockout (GnRH^hpg^) mice, which are deficient in circulating LH, make it apparent that LH plays a critical role in the development of ALCs. Lhb-null mice have decreased testes size, with very few ALC in the interstitial compartment, and with reduced serum testosterone levels (Ma et al., 2004). In the GnRH knockout mice, LC number is about 10% of control (Baker and O'Shaughnessy, 2001). Moreover, LH/hCG administration to GnRH knockout mice *in vivo* increases LC proliferation (Dombrowicz et al., 1992). In addition to the ligand, knockout of Lhcgr also significantly reduced ALC numbers (Lei et al., 2001; Zhang et al., 2001). Overall, these results suggest that LH signaling is critical to both ALC differentiation and their precursor proliferation. However, the specific mechanism that is responsible for its stimulation on Leydig cell proliferation is not well studied, though activation of ERK1/2 cascade may be involved (Ge and Hardy, 1997; Shiraishi and Ascoli, 2007).

### Androgen

Androgen receptor (NR3C4) is a nuclear receptor and is expressed in SLCs and the LC lineage and other testicular cells such as Sertoli cells and peritubular myoid cells (Bremner et al., 1994; Shan et al., 1995). The levels of NR3C4 are high in SLCs, PLCs and ILCs compared to ALCs (Shan and Hardy, 1992). The presence of NR3C4 in the LC lineage suggests that androgen produced by FLCs, adrenal glands or the LCs themselves might regulate the development and function of LCs. In NR3C4 mutated mice in which there is an androgen insensitivity, reduced differentiation of LCs and decreased LC numbers have been noted (O'Shaughnessy and Murphy, 1993; Murphy et al., 1994). Consistent with this, when PLCs are cultured *in vitro*, adding androgen greatly increased the efficiency of the cells to differentiate into androgen-producing cells (Hardy et al., 1990). All these evidences strongly suggest that androgen is essential for the development and maturation of ALC.

In mouse testis with global knockout of NR3C4, FLCs are normal while ALC number are reduced to 60% of control level by the adult, with very low expression of HSD17B3, suggesting that the differentiation of SLCs into ALCs is disrupted (O'Shaughnessy et al., 2002). Because NR3C4 is expressed by many testicular cell types, the result of global knockout, by itself, cannot tell which cell or cells are responsible for the ALC developmental defects. Recent studies, including cell-specific knockouts, demonstrated that NR3C4 in Sertoli cells (De Gendt et al., 2005; Hazra et al., 2013; Rebourcet et al., 2014a,b) and peritubular myoid cells (Welsh et al., 2009, 2012) are critical for ALC development. Interestingly, knockout of NR3C4 in Sertoli cells only reduced LC number but not the steroidogenic capacity (Chang et al., 2004). Sertoli cell specific NR3C4 deletion caused a significant downregulation of PDGF-AA, which, by reducing SLC/PLC proliferations, could be one of the mechanisms for the reduced ALC numbers (Chang et al., 2004).

To understand whether NR3C4 signaling in LCs also affect their own development, LC-specific knockout was attempted by using Amhr2-Cre (Xu et al., 2007). Though Amhr2-Cre was unable to produce a complete knockout in LCs and plus the gene is also disrupted in some Sertoli cells, the experiment indeed showed that the differentiation of LC is delayed (Xu et al., 2007). Fatty acid binding protein 4 (Fabp4)-Cre was also used to produce LC-specific knockout, which though only yielded disruptions in about 75% LCs, they were LC-specific. The results indicated that NR3C4 in LCs is indeed important for ALC maturation (O'Hara et al., 2014). mFLE-Cre was used to produce NR3C4 knockout in FLCs which, as expected, did not affect FLCs. Also, testosterone levels and fertility were normal in the adult mice, suggesting that NR3C4 signaling in FLCs is dispensable for male reproductive functions in the late life (Shima et al., 2015). However, in another study that NR3C4 is disrupted in the fetal testis or androgen level is reduced by the period resulted in a significantly reduction in SLC number (by 40%), and led to a significantly lower ALC numbers by the adult, suggesting that androgen proflies in fetal period is still important to the development of ALC in the late life (Kilcoyne et al., 2014).

### Notch

Notch signaling plays a critical role in determining cell fate and maintaining stem cells numbers (Lai, 2004). Four Notch receptors (Notch1–Notch4), the transmembrane receptors, can bind to multiple Notch ligands, including delta-like 1, delta-like 3, delta-like 4, jagged 1, and jagged 2. Binding the ligands activates γ-secretase-dependent proteolysis within the transmembrane domain to release the Notch intracellular domain (De Strooper et al., 1999; Huppert et al., 2000). The domain is then translocated to the nucleus where it binds DNA in a complex with an obligate co-factor, RBPJK (Jarriault et al., 1995), activating the transcription of its downstream target effectors, the HES and HEY proteins. The HEY and HES proteins are two families of basic helix-loop-helix Orange transcriptional repressors (Nakagawa et al., 2000).

Notch signaling pathway may play an important role in LC development. Notch signaling proteins are expressed in the fetal testis, ILCs and ALCs (Tang et al., 2008). Activation of Notch signaling in gonadal SLCs dramatically reduces FLC numbers (Tang et al., 2008). Conditional deletion of jagged 1 (a Notch ligand) in the interstitial cells leads to a significant increase in ALC numbers (Defalco et al., 2013). Activation of Notch signaling represses the GATA4-related promoter activity which may inhibit the expression of steroidogenesis-related genes (*Star* and *Hsd3b*) (George et al., 2015). Blocking Notch signaling by a γ-secretase inhibitor DAPT, can significantly promote the differentiation of SLCs into ALCs in a rat seminiferous tubule culture system (Li et al., 2016).

### Other factors

In addition to the factors discussed above, there are other factors that may also have potential effects on the LC development, although the *in vivo* evidences or specific mechanistic studies for these factors are lacking.

In addition to androgen, estrogen may also affect LC development. Two types of estrogen receptors α and β have been identified. Estrogen receptor α and β are detected in mouse and rat LCs (Zhou et al., 2002; Akingbemi et al., 2003). Estrogen receptors α is also found in the LC lineage (Zhou et al., 2002; Akingbemi et al., 2003). In the estrogen receptor knockout mice, LC steroidogenic capacity is increased relative to wild-type controls (Akingbemi et al., 2003). However, since estrogens are capable of affecting pituitary LH secretions by a negative feedback mechanism, these *in vivo* results cannot distinguish a direct effect of estrogen from an indirect effect from the pituitary LH (Akingbemi et al., 2003), though there is *in vitro* evidence to suggest the former. For example, estrogen seems able to bind G protein-coupled receptor 30 and inhibit LC steroidogenesis (Vaucher et al., 2014).

Epidermal growth factor (EGF) family contains seven ligands: EGF, transforming growth factor-α (TGFA), heparin-binding EGF-like growth factor, betacellulin, amphiregulin, epiregulin, and epigen (Singh et al., 2016). They bind to a family of receptors including the EGF receptor (EGFR), HER2, HER3, and HER4 (Singh et al., 2016). EGF is able to stimulate rat ILC proliferation via the phosphorylation of ERK1/2 and Akt (Tai et al., 2009). EGF is also capable of stimulating steroidogenesis of ALCs (Verhoeven and Cailleau, 1986). Another way that EGF signaling can affect Leydig cells is that the receptor can be trans-activated by LH/cAMP/PKA signaling which can further induce the subsequent activation of mitogen-activated protein kinase (MAPK), ultimately leading to StAR phosphorylation and mitochondrial translocation (Evaul and Hammes, 2008).

TGFA is another member of the EGF family. TGFA is present in the rat LC lineage, from as early as the progenitor stage (Teerds et al., 1990). TGFA stimulates PLC proliferation like EGF (Khan et al., 1992b) and increases the androgen production, as well as the expression levels of *Cyp17a1, Star*, and *Scarb1*, suggesting that it can function as a positive regulator in ILC development (Millena et al., 2004).

Wnt4 is a member of the Wnt family. Wnt4 binds to Frizzled cell-surface receptors to activate signaling cascades to eventually affect gene expressions. Overexpression of Wnt4 disrupts the formation of FLCs and thus the androgen production, possibly by affecting the migration of SLCs into the testis cord (Jeays-Ward et al., 2003; Jordan et al., 2003). The activation of Wnt signaling leads to the inhibition of GSK3β (Valvezan and Klein, 2012). Indeed, *in vitro* experiment indicated that the metal lithium, which inhibits GSK3β, robustly stimulated SLC and PLC differentiation (Li et al., 2016).

Interleukins (including interleukin-1, 2, and 6) are produced by testicular macrophages. They may play a role in LC development since deletion of testicular macrophages dramatically delayed the regeneration of LCs in the ethane dimethane sulfonate-treated model (Gaytan et al., 1994). *In vitro*, interleukin-1β (IL1) stimulates the mitosis of PLCs (Khan et al., 1992a). Interleukins are certainly a group of molecules that have potentials to influence LC development and functions, though there is still debate about their specific effects on steroidogenesis (Calkins et al., 1988; Guo et al., 1990; Hales, 1992).

### Role of sertoli cells

Sertoli cells play a very critical role in the development of LCs. When ILCs were co-cultured with Sertoli cells, they produced much higher amount of androgens and developed more abundant smooth endoplasmic reticulum (Tabone et al., 1984; Perrard-Sapori et al., 1987). Deletion of Sertoli cells during the neonatal and adult period caused significant reductions in PLC and ALC numbers (Rebourcet et al., 2014b). However, deletion of Sertoli cells during the fetal period seems not affecting FLCs (Rebourcet et al., 2014b). As shown above, specific knockout of NR3C4 in Sertoli cells significantly affected ALC development, indicating that NR3C4 may play an important role in regulating Sertoli cell paracine factors that may be critical for ALC development and functions.

## Summary

The postnatal development of ALCs begins with a pool of SLCs. Several pieces of evidence suggest that SLCs are multipotent and are able to differentiate into different types of cells, in addition to cells in Leydig lineage. It has been shown that SLCs are able to self-renew indefinitely, differentiate and thus commit to the LC lineage, and replenish their niche in the testes. After SLC commitment into Leydig lineage, there are two distinct intermediate stages before the cells matured to ALC. The first stage is represented by spindle-shaped PLC that expresses the steroidogenic enzymes CYP11A1, HSD3B, and CYP17A1 and then followed by the second stage, the ovoid-shaped ILCs that expresses HSD17B3 and HSD11B1. The transitions from SLC to PLC and ILC and finally to ALC are regulated by multiple hormones (LH and androgen) and growth factors (leukemia inhibitory factor, Desert Hedgehog, Platelet-derived growth factors, Kit ligand, insulin-like growth factor 1, fibroblast growth factor 2, notch ligands, and transforming growth factors, etc.). The understanding of the development of SLCs into the LC lineage will surely help us to better understand the development of male reproductive system and the mechanisms behind some of the testicular dysgenesis syndrome and hypogonadism, as well as how environmental factors may affect the male reproductive system. There are still plenty unknowns about SLCs and their regulation. The studies of SLCs themselves could benefit the regenerative medicine that the hypogonadism and infertility could be managed with stem cell solutions someday.

## Author contributions

LY and RG wrote the review. XL prepared tables. LL prepared figures. HC reviewed the manuscript.

### Conflict of interest statement

The authors declare that the research was conducted in the absence of any commercial or financial relationships that could be construed as a potential conflict of interest.
